# Bridging the gap: factors influencing the willingness-behavior transformation of older adults in using community care services in urban China

**DOI:** 10.3389/fpubh.2025.1664071

**Published:** 2025-10-15

**Authors:** Liu Yang, Lijian Wang

**Affiliations:** School of Public Policy and Administration, Xi’an Jiaotong University, Xi’an, China

**Keywords:** community care services, willingness - behavior transformation, influencing factors, older people, China

## Abstract

**Objectives:**

While the potential benefits of community care services (CCSs) have been widely recognized, a significant imbalance remains between the high demand for and the low utilization of these services among older adults in China. Therefore, this study aimed to identify key factors influencing the willingness-behavior transformation of older adults to use CCSs, thereby promoting the actual usage and improving their quality of life.

**Methods:**

Using survey data from 1,233 older adults in urban areas across 4 provinces in China, descriptive statistics analysis was conducted to illustrate the basic characteristics of participants, as well as their willingness and willingness-behavior transformation regarding the use of CCSs. Binary logistic regression and multinomial logistic regression models were used to explore the factors influencing both willingness and the willingness-behavior transformation of older adults in using the three CCS categories.

**Results:**

The willingness of the older adults to use CCSs was high, while the willingness-behavior consistency was relatively low, indicating the great gaps between positive willingness and actual behavior. The willingness of the older adults to use 3 CCS categories was significantly and differently influenced by 13 predisposing factors, enabling factors, and needs factors. Among them, age, sex, marital status, educational level, traditional old-age care belief, neighbor’s attitude, policy advocacy, service affordability, province, health status, and empty nest were identified as the key factors to convert the willingness into behavior.

**Conclusion:**

This study highlights key factors to willingness and willingness-behavior transformation for older adults to use CCSs in urban China, and further provides policy implications for government measures to incentivize the older adults’ positive willingness to use CCSs and bridge the gaps between the willingness and the actual behavior.

## Introduction

The rapidly increasing demand for social care services driven by a large aging population has raised significant concern worldwide, and China is no exception (1). As a country deeply influenced by the traditional culture of filial piety, China has long prioritized family care as the primary means of meeting the care needs of older adults (2, 3). However, with the rapid industrialization and a declining fertility rate, traditional family-based care in China has been challenged, and the pressure of elder care is shifting from the family to society. Compared with the rapid population aging taking place in China in the past 20 years, the accessibility of institutional care resources has not improved correspondingly (4), with only a minority of older adults able to afford this limited care resource. Meanwhile, like the older adults in most countries, Chinese seniors prefer to age in place rather than age in institutions, which has been proven to be effective in improving the quality of life (5, 6). Therefore, the Chinese government has put great effort into the development of community care services (CCSs) for the aged to support them in aging.

CCSs refer to formal care services for the community-dwelling older adults in the form of visiting services or concentrated services in community centers, which are supported by the government and provided by professional service institutions (7, 8). These institutions are screened by the community and basic-level government according to their quality and assessed according to the demands of the older adults. In fact, the Chinese government had put forward the preliminary concept of providing care support for the aged based on the community as early as the end of the last century (9). In 2011, the strategy of developing CCSs was formally laid out by the Chinese government (10, 11). During the period of the 13th Five-Year Plan (2016–2020), the provision capacity and coverage of CCSs have tremendously improved. By 2020, 203 cities, covering 60% prefecture-level cities in China, have been selected as pilot districts to implement the CCS policies (12). The number of community care centers has increased from 111 thousand in 2016 to 291 thousand in 2020, increasing by 162% (13), and the percentage of the community-dwelling older adults covered with these facilities has increased from 26.10% in 2008 to 62.02% in 2018 (14).

Ideally, CCSs are expected to help older adults prevent a decline in functional capacity and remain independent, thus reducing the pressure on family caregivers and meeting the preference for aging in place (15). With these benefits, Chinese seniors have shown a surprisingly high demand for CCSs. Data from the Chinese Longitudinal Healthy Longevity Survey (CLHLS) indicate that the percentage of older adults wanting to use CCSs in 2011, 2014, and 2018 was 90.34, 88.93 and 89.39%, respectively, in China (14). However, in contrast to the stubbornly high demand and constantly increasing provision, the under-utilization of CCSs has been a long-standing issue in China. According to China Longitudinal Aging Social Survey (CLASS) data, the percentage of surveyed older adults who had used CCSs in 2014, 2016, and 2018 was only 6.96%, 6.86%, 9.97%, respectively, far below the rates of demand and supply. The great gap between demand and utilization of CCSs runs contrary to the government’s wishes and reflects the huge waste of public resources. In fact, it is a typical superficial form of theoretical “say one thing” rather than practical “do” (16), which raises our concern about what factors evoke older adults’ willingness to use CCS and how to cross the chasm from willingness to behavior.

Some studies have investigated the determinants of older adults’ willingness (17) or behavior (18) to use CCS, though few of them have noticed the transformation of willingness and behavior. The theory of planned behavior suggests that willingness is an important predictor of behavior, while not all willingness can transform into behavior due to the influence of time, behavioral capacity, or environmental constraints (19). Therefore, using willingness as the dependent variable to identify potential predictors may exaggerate their influence on actual behavior (20, 21), potentially leading to decision-making biases in government policy related to people’s behaviors (22).

In terms of studies on factors influencing older adults’ willingness or behavior to use CCS, scholars have provided some empirical evidence and made substantial progress. Previous studies have introduced Anderson’s behavioral model as a concept framework to select the potential influencing factors, including three sets of factors: need factors, predisposing factors, and enabling factors (18, 23, 24). Needs factors, usually measured by health situation or functional limitation, were evidenced as the core driving factors of using CCSs in most existing studies (25–27). Personal demographic and sociological characteristics such as age, sex, education, or marital status were identified as predisposing factors in previous studies, as they may influence an individual’s propensity to use CCSs (28, 29). Enabling factors were selected from the aspects of personal or family financial capability and service accessibility to explore the driving force of using CCSs (7, 30). Similar to many other Asian countries, China has a profound familism culture and a traditional belief in raising children for old age, which fosters a strong reliance on family care and limits acceptance of social care services (31). According to Anderson’s behavioral model, individual beliefs are also a key component of predisposing factors (32), although this aspect has been overlooked in most studies. Moreover, previous studies suggested that with self-care capacity and function declining, the informal care support from a spouse or adult children is the primary choice of older adults (33). When family support is absent or inadequate for meeting their demands, they would further turn to social care services for help (34, 35). Therefore, lacking family care may be a non-negligible part of the needs factor. Overall, previous literature suggested that predisposing factors and enabling factors had a significant impact on older adults’ willingness or behavior to use CCSs, while results were varied from countries or regions with different economic and social environments (18, 36). Hitherto, some studies have been conducted in China on the factors associated with using CCSs, while most of them focused on urban cities, especially the front-runner cities of the national economy and public resources (37), which might lead to some applicability bias in these results.

Therefore, the objectives of this study are to (1) reveal the gaps between willingness and behavior of older adults to use CCSs; (2) identify the key factors influencing older adults’ willingness to use CCSs; and (3) explore the incentives and barriers for older adults to convert their willingness into actual behavior regarding using CCSs. The results of this study would offer empirical evidence for policy-makers to formulate effective and suitable measures to promote CCS use among community-dwelling older adults and help them benefit from using CCSs sustainably.

### Theoretical framework

**Figure 1:**
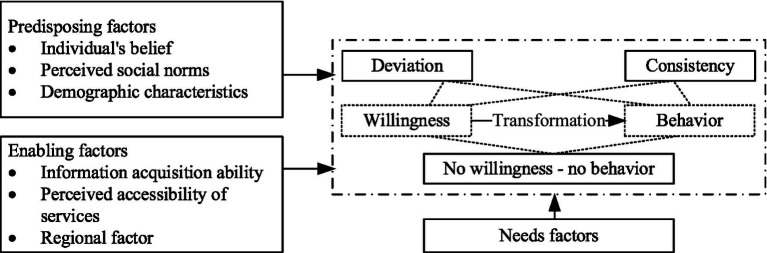
The theoretical framework of factors influencing willingness-behavior transformation of older adults to use CCSs.

Anderson’s behavioral model and the theory of planned behavior (TPB) are widely used to identify the factors associated with older adults’ willingness and behaviors to use care services. The TPB indicates that willingness is the direct predictor of individual behavior decisions, which are influenced by attitude, subjective norms, and perceived behavioral control. As a mature analytical framework with a convincing interpretation, Anderson’s behavioral model suggests that the use of medical care services is affected by needs, predisposing, and enabling factors. In this study, the TPB and Anderson’s behavioral model were integrated to form our conceptual framework (see Figure 1).

Specifically, with regard to the three sets of influencing factors in Anderson’s behavioral model, the central role of the care needs in driving CCS utilization has been evidenced in previous studies (38). Furthermore, the predisposing factors, like demographic characteristics and individual beliefs, can affect people’s propensity to use social care (18). Among them, an individual’s belief and the attitude of the TPB were merged as they have essential consistency, which reflects one’s positive or negative belief or attitude toward some behaviors and greatly determines their actual behaviors (19). In addition to the individual-level attitude, the TPB indicates that social norms, reflecting the attitude or belief from the environmental level, such as family, neighborhood, and society, to CCS may also affect one’s willingness and behaviors. Hence, we integrated social norms into the predisposing factors. Enabling factors reflect the capability of individuals or families to obtain care service resources, including personal factors such as information acquisition ability, financial factors such as incomes, and community factors such as the accessibility of services in each community. Similar to the perceived behavioral control of the TPB, which indicates the facilitative or suppressive factors perceived by people in acting on a behavior (39), enabling factors could form individuals’ positive perception and expectation of CCS utilization and further facilitate their behaviors.

All influencing factors are expected to predict the willingness of older adults to use CCSs and further impact the use behavior. For the transform status between positive willingness and actual behavior, three possible results are expected, including willingness-behavior consistency, willingness-behavior deviation (both willingness-no behavior and no willingness-behavior), and no willingness-no behavior.

## Materials and methods

### Sampling and data collection

A cross-sectional survey was conducted by our research team from January to April 2023 in China. Four provinces, namely Jiangsu, Hebei, Hubei, and Shaanxi, were included in this survey. These provinces cover the eastern, western, and middle regions of China. In terms of CCS development, the Chinese government has started promoting the establishment of CCS facilities since 2010 with policy and financial support. During the period of the 13th Five-Year Plan (2016–2020), the national CCSs pilot districts selected from Jiangsu, Hubei, Shaanxi, and Hebei were 10, 9, 8, and 4, ranking at the upper-middle level among 30 provinces (40). By the end of 2023, the facility coverage rate of CCSs in all four provinces has reached 90%, making it reasonable to choose them as an epitome of the general development status of CCSs in China.

The survey was fully conducted using the stratified sampling method. In each province, according to the administrative division and economic development level, three cities were selected, and the isometric random sampling method was used in the selection of two or three counties (districts) in each city. Three to four typical urban communities equipped with integrated service centers of CCSs were selected according to their administrative divisions. In each community, about 30 participants were randomly selected with the help of grassroots community workers. Specifically, the older adults were invited to participate in our survey if they (1) were aged ≥60 years, (2) were able to communicate easily or communicate with the investigators’ assistance, and (3) volunteered to participate in the study. Data collection took approximately 30 min for each participant through face-to-face interviews and surveys. A total of 1,233 older adults were included in the final sample. Informed consent was obtained from all participants prior to the interviews. Study protocols and consent forms were approved by the medical ethics committee of Health Science Center of Xi’an Jiaotong University (approval number 2016–416). All participants provided informed consent to participate in the study.

### Dependent variables

In this study, our dependent variables are the willingness and the willingness-behavior transformation of the older adults to use CCSs, which describe the matching status of the older adults’ willingness and behavior. Guided by the policies and previous literature, three CCS categories, including daily care services (DCS), health care services (HCS), and recreational and cultural services (RCS), were analyzed in this study. In terms of measurement, the willingness of the older adults to use each CCS category was measured by the question “Did you have the willingness or demand for using DCS/HCS/RCS in the past year?,” with 1 for “Yes” and 0 for “No.” Also, participants were asked if they used DCS/HCS/RCS in the past year to measure the behavior of the older adults to use each CCS category. Consequently, if the participant reported that he/she had the willingness or demand for one CCS category and had used it before, that is, he/she had both positive willingness and actual behavior, the consequence of willingness-behavior transformation was identified as “willingness-behavior consistency,” with a value of 2. If the participant reported that he/she had the willingness or demand for one CCS category but had never used it before, that is, he/she had only positive willingness, the consequence was identified as “willingness-behavior deviation,” with a value of 1. If the participant reported that he/she had no willingness or demand for one CCS category and had never used it before, the consequence was identified as “no willingness-no behavior,” with a value of 0. Another possible matching status was “no willingness-behavior,” in which a participant reported no willingness or demand for a particular CCS category, but had previously used it. However, since no participants reported this status in the survey, it was not included in the discussion of this study.

### Independent variables

Following the conceptual framework proposed above, three sets of potential influencing factors were identified. Predisposing factors include demographic and socioeconomic characteristics, traditional older adults care beliefs, and perceived social norms. Demographic characteristics were measured by age, sex, marital status, and education level. Traditional old-age care belief (TOCB) may affect an individual’s attitude to use CCSs, which was measured by the question “Do you agree with the concept of raising children for old age,” with 1 for “Yes” and 0 for “No.” Perceived social norms include three variables of “family’s attitude,” “neighbor’s attitude,” and “policy advocacy.” Family’s attitude was measured by the item “My family members support me to use CCSs,” with 1 for “Support” and 0 for “Non-support.” Neighbors’ attitude and policy advocacy were measured by items “Older people around me often use CCSs” and “I often hear the policy advocacy about CCSs,” rated on a five-point Likert scale from very disagree = 1 to very agree = 5.

Enabling factors include an individual’s information acquisition ability (IAA), space accessibility, service affordability, and province, reflecting the accessibility of CCSs to participants. IAA and space accessibility were measured by items “I can use the Internet to search information of CCSs” and “It is convenient to get to the CCSs center from my home,” with 1 for “Yes” and 0 for “No.” Service affordability was measured by the item “The price of DCS/HCS/RCS is reasonable,” with responses ranging from disagree = 1 to very agree = 5. Given the influence of the development levels of CCSs and the economy on the social care resources, the province was measured as a regional variable in this study.

Health status and family care status were selected as needs factors. Health status was measured by the question “How do you rate your health status?” which is rated on a three-point Likert scale with bad = 1, fair = 2, and good = 3. Family care status was reflected by asking participants if they were an empty nest, with 1 for “Yes” and 0 for “No.” The detailed coding and descriptive statistics of each set of variables are shown in Table 1.

**Table 1:** Descriptive statistics of influencing factor variables.

Variables		Definition/Code	N (%)/Mean (SD)
Predisposing factors	Age	Continuous variable	71.41 (7.84)
	Sex	1 = Female	630 (51.09)
		0 = Male	603 (48.91)
	Marital status	1 = Single	340 (27.58)
		0 = Married	893 (72.42)
	Education level	1 = Primary school and lower	557 (45.17)
		2 = Middle school	341 (27.66)
		3 = High school and higher	335 (27.17)
	TOCB	1 = Yes	525 (42.58)
		0 = No	708 (57.42)
	Family’s attitude	1 = Support	549 (44.53)
		0 = Non-support	684 (55.47)
	Neighbor’s attitude	Continuous variable	3.31 (0.95)
	Policy advocacy	Continuous variable	2.91 (1.16)
Enabling factors	IAA	1 = Yes	1,008 (81.75)
		0 = No	225 (18.25)
	Space accessibility	1 = Yes	969 (78.59)
		0 = No	264 (21.41)
	Service affordability	Continuous variable	1.95 (0.71)
	Province	1 = Hebei	260 (21.09)
		1 = Jiangsu	242 (19.63)
		1 = Hubei	405 (32.85)
		1 = Shaanxi	326 (26.44)
Needs factors	Health status	Continuous variable	1.86 (0.87)
	Empty nest	1 = Yes	846 (68.61)
		0 = No	387 (31.39)

TOCB, traditional old-age care belief; IAA, information acquisition ability.

### Analytical strategies

In this study, descriptive statistics analysis and regression analysis are used. First, simple descriptive statistics are estimated to illustrate the basic characteristics of participants and their willingness and willingness-behavior transformation to use CCSs. Second, for the dependent variable of “willingness,” a binary logistic model was used to investigate the influencing factors of the older adults’ willingness to use CCSs. The model estimated by the Equation 1 is as follows:


(1) $$Y_{i}=\ln(\frac{p_{i}}{1-p_{i}})=\alpha +\sum_{n=1}^{n}\beta_{n}X_{\mathrm{ni}}$$

where Y denotes the probability of the older adult’s willingness to use CCSs. p represents the probability of the occurrence of willingness. β is the estimated coefficient. α is a constant term. X signifies the factor influencing the older adult’s willingness to use CCSs.

Thirdly, as the dependent variable of “willingness-behavior transformation” is a probability event, with three probable states of “willingness-behavior consistency (Y = 2),” “willingness-behavior deviation (Y = 1),” and “no willingness-no behavior (Y = 0),” the multinomial logistic regression models were used to further explore the factors influencing the willingness-behavior transformation of older adults to use CCSs. The model constructed in the Equation 2 is as follows:


(2) $$P_{i}(Y=i|X)=\frac{\exp(-\alpha_{i}+\sum_{j=1}^{n}\beta_{j}X)}{1+\exp(-\alpha_{i}+\sum_{j=1}^{n}\beta_{j}X)}$$

where P denotes the probability of the older adult’s willingness-behavior transformation and $P_{1}+P_{2}+P_{3}=1$ . β is the coefficient of the independent variable. α is a constant term. X is the set of influencing factors.

## Results

### Characteristics of participants

As shown in Table 1, a total of 1,233 older adults participated in the final survey. The demographic characteristics of participants are reported as follows: among them, 42.58% held the traditional belief in raising children for old age, and 44.53% received support from family members to use CCSs. The mean scores of neighbors’ attitude and policy advocacy were 3.31 and 2.91, respectively. Additionally, 81.75% of participants reported being able to use the Internet to search for CCS information. A total of 78.59% indicated that it is convenient to access the CCS center, and the mean score for service affordability was 1.95. The mean value of self-reported health status was 1.86, and 68.61% of participants were classified as empty-nest older adults.

### Willingness and willingness - behavior transformation of older adults to use CCSs

Table 2 shows the proportion of willingness and the three willingness-behavior transformation statuses of older adults to use CCSs. As a whole, participants showed high willingness, high willingness-behavior consistency, and high willingness-behavior deviation regarding using CCSs. 68.69% of participants showed positive willingness or demand for one or more CCSs. Among them, 33.98% of participants converted their willingness into actual behavior, while 34.71% of them had only willingness and failed to convert it into behavior.

**Table 2:** Descriptive statistics of willingness and willingness-behavior transformation.

Willingness and behavior	DCS		HCS		RCS		CCSs	
	*N*	%	*N*	%	*N*	%	*N*	%
Willingness	595	48.26	590	47.85	447	36.25	847	68.69
Willingness-behavior consistency	231	18.74	142	11.52	216	17.52	419	33.98
Willingness-behavior deviation	364	29.52	448	36.33	231	18.73	428	34.71
No-willingness-no behavior	638	51.74	643	52.15	786	63.75	386	31.31

In terms of the specific CCS category, the willingness of older adults to use CCSs showed a significant decrease. Participants having a willingness to use DCS, HCS, and RCS were 48.26, 47.85, and 36.25%, respectively. This illustrates that participants might show different willingness-behavior transformation status when using different CCS categories. Specifically, participants showing willingness - behavior deviation of using DCS, HCS, and RCS were 29.52, 36.33 and 18.73%, while participants showing willingness - behavior consistency were only 18.74, 11.52 and 17.52%, respectively, indicating the large gaps between high willingness and low behavior of older adults to use CCSs.

### Factors influencing the willingness of older adults to use CCSs

The binary logistics regression estimation was conducted to identify factors affecting the willingness of older adults to use DCS, HCS, and RCS. As shown in Table 3, except for age, all predisposing factors, enabling factors, and need factors had significant, albeit selective, effects on willingness to use CCS categories.

**Table 3:** Factors associated with the willingness of older adults to use CCSs.

Variables	DCS	HCS	RCS
Age	0.05	0.01	−0.05
	(0.07)	(0.07)	(0.07)
Female	0.37***	0.16	0.05
	(0.14)	(0.13)	(0.14)
Single	0.30*	−0.21	−0.22
	(0.16)	(0.15)	(0.16)
Middle school	0.25	−0.13	0.07
	(0.16)	(0.15)	(0.16)
High school and higher	0.56***	0.26	0.46***
	(0.18)	(0.16)	(0.17)
TOCB (Yes)	−0.38***	0.21	0.05
	(0.14)	(0.13)	(0.14)
Family’s attitude (Yes)	0.64**	0.29	0.41
	(0.27)	(0.25)	(0.26)
Neighbor’s attitude	1.98***	2.53***	2.37***
	(0.31)	(0.30)	(0.31)
Policy advocacy	0.49***	0.13**	0.25***
	(0.07)	(0.07)	(0.07)
IAA (Yes)	0.09	0.27*	−0.22
	(0.17)	(0.16)	(0.17)
Space accessibility (Yes)	0.34*	0.44**	−0.06
	(0.18)	(0.17)	(0.18)
Service affordability	0.35***	−0.07	0.32***
	(0.11)	(0.10)	(0.10)
Jiangsu	1.27***	0.93***	0.44*
	(0.25)	(0.23)	(0.24)
Hubei	0.17	−0.02	−0.17
	(0.26)	(0.24)	(0.25)
Shaanxi	−0.46	−0.68**	−0.50
	(0.31)	(0.29)	(0.31)
Health status	−0.21***	−0.12*	0.12
	(0.08)	(0.07)	(0.08)
Empty nest (Yes)	0.42***	0.10	0.23*
	(0.14)	(0.13)	(0.13)
Constant	−2.52***	−1.58***	−2.76***
	(0.35)	(0.32)	(0.35)
Observations	1,233	1,233	1,233

Standard errors in parentheses. ***p < 0.01; **p < 0.05; *p < 0.1.

Specifically, among the predisposing factors, female older adults were more willing to use DCS compared to their male counterparts. Participants who were single showed a higher willingness to use DCS than those who were married. Older adults who were educated in high school and higher had a significantly higher willingness to use DCS and RCS. Individuals agreeing with the traditional old-age care belief were more likely to depend on family care in daily life, thus significantly restraining their demands for DCS supported by the community. As for the social norms factors, family support had a positive effect on willingness to use DCS, and both the peer effect from the neighborhood and the guidance of policies had a significantly positive relationship with older adults’ willingness to use DCS, HCS, and RCS. For enabling factors, IAA had a positive and significant effect on willingness to use HCS, space accessibility was positively related to willingness to use DCS and HCS, and service affordability was positively associated with willingness to use DCS and RCS. Compared to participants living in Hebei, participants living in Jiangsu were more likely to use three categories of CCSs, while participants living in Shaanxi showed lower willingness to use HCS. Regarding needs factors, older adults with better health status showed less willingness to use DCS and HCS, and people who were empty nesters were more willing to use DCS and RCS.

#### Factors influencing willingness - behavior transformation of older adults to use CCSs

While factors influencing older adults’ willingness to use DCS, HCS, and RCS were identified, key factors promoting or impeding the willingness to convert into actual behavior were still unknown. Hence, Table 2 shows the results of multinomial logistic regression models on factors associated with willingness-behavior transformation of older adults to use DCS, HCS, and RCS.

In terms of predisposing factors, compared to the no-willingness-no-behavior group, all demographic factors had selectively significant associations with willingness-behavior consistency and willingness-behavior deviation of using different categories of CCSs. Additionally, TOCB was negatively associated with the willingness-behavior consistency of using DCS and positively associated with only the willingness to use HCS. For the social norm factors, family support had a positive effect on willingness to use RCS, but failed to convert the willingness into behavior. Both neighbors’ attitude and policy advocacy had positive relationships with willingness-behavior consistency regarding using three CCS categories.

In terms of enabling factors, compared to the no willingness - no behavior group, IAA was only positively related to the willingness-behavior deviation of using HCS, suggesting that older adults who were able to acquire community-based medical information via the Internet were more willing to use HCS, but it failed to convert the willingness into behavior. Furthermore, space accessibility had a positive association with willingness-behavior deviation of using DCS and HCS. As for service affordability, it had a positive effect on increasing older adults’ willingness to use DCS and converting it into behavior, while it only had a positive relationship with willingness-behavior deviation of using RCS. As for the regional factor, compared to Hebei province, older adults in Jiangsu province showed a higher willingness to use three categories of CCSs, but failed to convert it into behavior. Additionally, older adults living in Shaanxi showed significantly lower willingness and behavior consistency to use HCS.

In terms of needs factors, compared to the no-willingness-no-behavior group, health status was negatively related to willingness to use DCS and HCS, and positively converted the willingness to use RCS into behavior. Furthermore, an empty nest was evidenced to have a positive relationship with willingness to use RCS, and has a positive relationship with both willingness and behavior of using DCS.

#### Robustness tests

To ensure the reliability of the results, a series of robustness tests was conducted in this study. We changed the measurement of independent variables on the basis of the original models. Specifically, five continuous variables, including age, neighbor’s attitude, policy advocacy, service affordability, and health status, were remeasured by constructing an ordered categorical variable (age) and four dummy variables. Additionally, we changed the regression model to identify factors influencing willingness to use CCSs, and the binary logistics regression model was changed into the probit model. The robustness test results are presented in Table 5.

The results showed that there were minor differences in the values of coefficients, though the estimation results in Table 5 were basically consistent with those in Tables 3, 4. It indicated that the results on identifying factors influencing willingness and willingness-behavior transformation of older adults to use CCSs were robust and reliable in this study.

**Table 4:** Factors associated with willingness-behavior transformation of older adults to use CCSs.

Variables	DCS		HCS		RCS	
	Consistency	Deviation	Consistency	Deviation	Consistency	Deviation
Age	0.18*	−0.06	−0.03	−0.00	0.04	−0.12
	(0.10)	(0.08)	(0.11)	(0.07)	(0.09)	(0.09)
Female	0.62***	0.26*	0.09	0.10	0.08	0.02
	(0.21)	(0.15)	(0.22)	(0.14)	(0.18)	(0.17)
Single	0.78***	−0.02	−0.09	−0.24	−0.19	−0.22
	(0.22)	(0.18)	(0.25)	(0.16)	(0.20)	(0.19)
Middle school	0.01	0.33*	−0.19	−0.12	0.14	0.04
	(0.26)	(0.18)	(0.27)	(0.16)	(0.22)	(0.20)
High school and higher	0.88***	0.39**	0.64**	0.12	0.56**	0.34
	(0.25)	(0.20)	(0.27)	(0.18)	(0.22)	(0.21)
TOCB (Yes)	−0.75***	−0.14	−0.09	0.30**	0.14	−0.03
	(0.21)	(0.16)	(0.22)	(0.14)	(0.19)	(0.17)
Family’s attitude (Yes)	0.08	0.05	0.63	−0.32	−0.36	0.89***
	(0.40)	(0.28)	(0.39)	(0.25)	(0.37)	(0.34)
Neighbor’s attitude	4.91***	−0.04	4.52***	1.57***	3.07***	1.73***
	(0.48)	(0.38)	(0.47)	(0.33)	(0.40)	(0.38)
Policy advocacy	0.68***	0.42***	0.27**	0.09*	0.52***	0.06
	(0.11)	(0.08)	(0.12)	(0.05)	(0.10)	(0.09)
IAA (Yes)	0.31	0.11	−0.07	0.50***	−0.41	0.10
	(0.29)	(0.19)	(0.27)	(0.19)	(0.25)	(0.23)
Space accessibility (Yes)	0.04	0.43**	0.22	0.49***	0.04	−0.10
	(0.31)	(0.19)	(0.34)	(0.18)	(0.25)	(0.22)
Service affordability	0.57***	0.32***	−0.07	0.02	0.11	0.49***
	(0.17)	(0.11)	(0.17)	(0.10)	(0.14)	(0.13)
Jiangsu	0.61	1.31***	−0.69	1.13***	−0.14	1.09***
	(0.44)	(0.26)	(0.47)	(0.24)	(0.33)	(0.32)
Hubei	0.65	0.33	−0.51	0.41	−0.31	0.12
	(0.41)	(0.27)	(0.37)	(0.26)	(0.34)	(0.34)
Shaanxi	−0.26	0.30	−2.16***	0.33	0.01	−0.54
	(0.48)	(0.34)	(0.47)	(0.32)	(0.42)	(0.40)
Health status	0.04	−0.32***	0.20	−0.22***	0.34***	0.00
	(0.12)	(0.09)	(0.12)	(0.08)	(0.10)	(0.09)
Empty nest (Yes)	0.62***	0.35**	0.11	0.14	0.10	0.35*
	(0.23)	(0.16)	(0.24)	(0.14)	(0.20)	(0.19)
Constant	−6.61***	−1.70***	−3.95***	−1.79***	−3.45***	−3.94***
	(0.63)	(0.39)	(0.56)	(0.35)	(0.46)	(0.47)
Observations	1,233	1,233	1,233	1,233	1,233	1,233

Standard errors in parentheses. ***p < 0.01; **p < 0.05; *p < 0.1. No-willingness-no behavior group was treated as the reference group.

**Table 5:** Robustness tests on factors associated with willingness and willingness-behavior transformation to use CCSs.

Variables	DCS	HCS	RCS	DCS		HCS		RCS	
	Probit	Probit	Probit	Consistency	Deviation	Consistency	Deviation	Consistency	Deviation
70–79	0.11	−0.02	−0.04	0.32	0.05	0.07	−0.05	−0.21	0.05
	(0.09)	(0.08)	(0.09)	(0.21)	(0.16)	(0.23)	(0.15)	(0.19)	(0.17)
80+	0.16	0.11	0.01	0.81***	−0.10	0.27	0.14	0.15	−0.14
	(0.12)	(0.11)	(0.12)	(0.26)	(0.23)	(0.29)	(0.20)	(0.24)	(0.25)
Female	0.22***	0.06	0.04	0.56***	0.25*	0.10	0.11	0.07	0.02
	(0.08)	(0.08)	(0.08)	(0.19)	(0.13)	(0.21)	(0.14)	(0.18)	(0.17)
Single	0.17*	−0.10	−0.11	0.84***	−0.06	0.03	−0.24	−0.09	−0.22
	(0.09)	(0.09)	(0.09)	(0.20)	(0.18)	(0.23)	(0.16)	(0.20)	(0.19)
Middle school	0.15	−0.09	0.05	0.08	0.37**	−0.22	−0.10	0.11	0.08
	(0.10)	(0.09)	(0.10)	(0.24)	(0.17)	(0.26)	(0.16)	(0.22)	(0.19)
High school and higher	0.33***	0.15	0.30***	0.89***	0.50**	0.58**	0.14	0.58***	0.38
	(0.10)	(0.10)	(0.10)	(0.23)	(0.19)	(0.26)	(0.17)	(0.22)	(0.24)
TOCB (Yes)	−0.29***	0.06	−0.02	−0.97***	−0.18	−0.29	0.25*	0.00	−0.07
	(0.08)	(0.08)	(0.08)	(0.20)	(0.16)	(0.22)	(0.14)	(0.18)	(0.17)
Family’s attitude (Yes)	0.32*	0.18	0.24	0.41	0.35	0.30	0.02	−0.33	0.95***
	(0.16)	(0.15)	(0.16)	(0.40)	(0.30)	(0.43)	(0.27)	(0.37)	(0.36)
Neighbor’s attitude (Yes)	0.53***	0.62***	0.65***	1.69***	0.47**	1.85***	0.63***	1.33***	0.81***
	(0.10)	(0.10)	(0.10)	(0.26)	(0.19)	(0.26)	(0.18)	(0.23)	(0.21)
Policy advocacy (Yes)	0.26***	0.11***	0.32***	1.11***	0.39**	0.37***	0.12**	1.13***	0.03
	(0.04)	(0.03)	(0.09)	(0.20)	(0.17)	(0.10)	(0.06)	(0.19)	(0.19)
IAA (Yes)	0.12	0.18*	−0.13	0.05	0.11	−0.25	0.46**	−0.41	0.13
	(0.11)	(0.10)	(0.10)	(0.26)	(0.19)	(0.26)	(0.18)	(0.25)	(0.23)
Space accessibility (Yes)	0.19*	0.25**	−0.02	−0.13	0.37**	0.08	0.50***	0.04	−0.09
	(0.11)	(0.10)	(0.11)	(0.30)	(0.19)	(0.33)	(0.18)	(0.25)	(0.22)
Service affordability (Yes)	0.35***	−0.13	0.22**	0.92***	0.37*	−0.09	−0.20	−0.15	0.47**
	(0.10)	(0.10)	(0.10)	(0.27)	(0.20)	(0.26)	(0.18)	(0.23)	(0.23)
Jiangsu	0.82***	0.73***	0.48***	0.73	1.68***	−0.18	1.45***	0.25	1.37***
	(0.16)	(0.15)	(0.16)	(0.45)	(0.29)	(0.49)	(0.27)	(0.36)	(0.34)
Hubei	0.12	0.02	−0.10	0.57	0.13	−0.62	0.36	−0.29	0.04
	(0.16)	(0.15)	(0.15)	(0.40)	(0.28)	(0.40)	(0.27)	(0.33)	(0.34)
Shaanxi	−0.25	−0.33*	−0.22	−0.33	−0.02	−2.47***	0.23	0.15	−0.55
	(0.19)	(0.18)	(0.19)	(0.47)	(0.35)	(0.49)	(0.33)	(0.42)	(0.41)
Health status (Healthy)	−0.23***	−0.25***	0.13	−0.03	−0.50***	0.04	−0.55***	0.45**	0.04
	(0.08)	(0.08)	(0.08)	(0.19)	(0.15)	(0.21)	(0.13)	(0.18)	(0.16)
Empty nest (Yes)	0.26***	0.05	0.16*	0.80***	0.32**	0.21	0.06	0.17	0.36*
	(0.09)	(0.08)	(0.09)	(0.22)	(0.16)	(0.23)	(0.14)	(0.19)	(0.19)
Constant	−1.98***	−0.88***	−1.05***	−4.76***	−2.06***	−3.47***	−1.71***	−2.23***	−2.98***
	(0.19)	(0.18)	(0.17)	(0.47)	(0.32)	(0.49)	(0.32)	(0.36)	(0.38)
Observations	1,233	1,233	1,233	1,233	1,233	1,233	1,233	1,233	1,233

Standard errors in parentheses. ***p < 0.01; **p < 0.05; *p < 0.1. No-willingness-no behavior group was treated as the reference group.

## Discussion

By revealing the gap between willingness and behavior in older adults’ use of CCSs in urban China, this study analyzed the factors influencing their willingness to use CCSs and further identified the promoting and impeding factors affecting the transformation of willingness into actual behavior. The results showed that: (1) the willingness of the older adults to use CCSs was high, while the willingness-behavior consistency was relatively low, indicating the great gaps from converting positive willingness into actual behavior; and (2) a series of predisposing factors, enabling factors, and need factors were evidenced to have significant, albeit selective, impacts on arousing the willingness of the older adults to use three CCS categories. Among them, age, sex, marital status, educational level, traditional old-age care belief, neighbor’s attitude, policy advocacy, service affordability, province, health status, and empty nest were identified as the key factors to convert the willingness into behavior.

Specifically, the results suggest that age was an important predisposing factor evoking the older adults’ willingness to use DCS and further converting it into active behavior. Consistent with previous studies, the older adults in higher age groups have a higher risk of declining self-care ability, and they are more likely to receive home care services like help with bathing, meals, and housework (41). Compared to male older adults, female older adults had a higher willingness to use DCS and SCS Gender differences were revealed when receiving formal care services for older adults (42, 43). Single older adults were more willing to use DCS and converted their willingness into behavior. This finding was in line with previous research reporting that older adults with a spouse were less likely to be admitted into nursing homes and had lower needs for social care services. As the spouse is the important care provider in the family care mode, the result that single older adults tend to seek help for social care services is not surprising. For the positive effect of educational level on willingness and willingness-behavior consistency of using DCA, HCS, and RCS, the possible explanation is that the educated older adults may have stronger awareness and abilities to access and use social care services to improve their life quality (7). As expected, the older adults who did not stick to the traditional concept of relying on children for old age were more receptive to using DCS, which sheds light on the importance of belief in the choice of care mode. Additionally, our results suggest that neighbors’ attitudes and policy advocacy had a positive impact on the older adults’ willingness to use CCSs and could further enhance their behavior partially. Similarly, a study in Spain revealed that urban contexts (neighborhoods) are linked to the use of formal public services and private care at home (44), echoing the social ecosystem theory, which indicates that individuals’ behavior is deeply influenced by the family environment, peer effects from the neighborhood, and a positive policy environment (45).

Regarding the enabling factors, IAA only had a positive effect on the willingness to use HCS and failed to convert it into behavior. Theoretically, better IAA means higher accessibility to knowledge and information of CCSs, which are an important incentive for arousing interest in using CCSs, but not enough for determining their behavior decisions (39). Another possible explanation is that the relatively lower digital literacy of older adults may hamper their quality of information retrieval and further affect their perception of CCSs. Space accessibility could improve willingness to use DCS and HCS, which is consistent with similar research (46). The affordable price could improve willingness to use RCS and boost both willingness and behavior of the older adults to use DCS. In recent years, DCS such as different grades of canteen services for the older adults have been vigorously supported by the Chinese government with special policies, and a higher quality of DCS can attract the older adults with higher payment capacity (47). For the significant relationship between service affordability and DCS use, our findings are partially in line with a study conducted in Australia, which found that the cost of the service was a barrier to accessing services for older adults (48). However, consistent with an existing study reporting that the perceived low cost of community healthcare service had no significant impact on their choice (37), service affordability was not significantly related to HCS use in this study. A possible explanation is that the competencies of medical personnel and medical facilities are more important than low costs in choosing healthcare service providers. Moreover, the relationship between regional factor and CCSs use indicated that older adults in more developed region were more likely to use CCSs, while older adults in comparatively less-developed area showed significantly lower willingness and less behavior of using HCS, which might be attributed to the influence of regional economic development level on the accessibility of CCSs resources and the regional cultural on individuals’ and families’ attitude on social care.

As for needs factors, worse health status implies decreased physical function and self-care ability, and heavier daily care and medical care burdens on the family, thus increasing willingness for DCS and HCS. However, influenced by filial piety culture, family care remains the primary means of meeting the daily care needs of older adults (31), which may hinder the transformation of willingness to use DCS into actual behavior. Meanwhile, while the Chinese government has been working on strengthening the ability of the primary health care system based on the community, the great health resource inequality between community-level and higher-level health care facilities is objective, resulting in the low utilization of community health services (49). Additionally, given that good physical function is the foundation of activity participation, our results on the positive relationship between health status and willingness-behavior consistency of using RCS were not surprising. Consistent with our expectation, the older adults who were empty nesters showed a higher willingness to use DCS and RCS, as they had higher demands for social care support to fill the lack of family care and meet their emotional needs (50). Our findings echo suggestions from previous studies that older adults lacking family care were more likely to use DCS but not HCS. It suggested that the relationship between family care and CCSs is both mixed, whereas high-professional services like HCS are a supplement for family care, and non-professional services such as DCS may be a substitute for family care (31, 51). Whatever the development of CCSs, the role of the family in improving the quality of life of most Chinese seniors is indispensable, and it should give greater attention to the family in Chinese community care policy and summon up more family support.

### Policy implications

Based on these findings, effective policy implications to improve the willingness and bridge the gap between willingness and behavior can be put forward. First, the exact demand evaluation is key to the effective supply of CCSs and transforming the older adults’ willingness to use CCSs into behavior. During the evaluation, more attention should be paid to the differential characteristics of older adults to better match their service demands. For instance, the age structure of the community should be taken into consideration in the allocation of DCS resources, with more resources allocated to the advanced aging communities. Furthermore, people-targeted services such as bath assistance and housework support need to be designed for older adults who are female, single, or in an empty nest. High-quality medical services and diverse recreation service items should be improved to meet the high demands of the educated older adults.

Second, in addition to guiding the older adults’ beliefs and evoking their potential demands for CCSs, more efforts are needed to create a positive and supportive environment, especially the neighborhood and policy environment, for them to use CCSs. For instance, it is important to strengthen the promotion of CCSs’ merits in improving quality of life in later years and alleviating the burden of family care. Policy guidance should also be directed toward community-dwelling older adults with care needs, as well as their family members. Additionally, as neighborhood welfare participation plays an important role in an individual’s welfare participation (52), it is suggested to give full play to the positive neighborhood effect and peer effect of using CCSs. The key is to take improving service ability and users’ satisfaction as the core and further establish community user mutual assistance groups to take these users as important propagandists to mobilize more people.

Third, it is necessary to take measures to improve the accessibility of CCSs for community-dwelling older adults. There has been a wide consensus that Internet technology has great application prospects in widening public resource access (18). Therefore, extending the Chinese government’s ‘Internet Plus’ strategy into the field of CCSs is urgently needed, especially through the application of Internet technology in the demand collection and service delivery. This would help improve the matching of supply and demand and eliminate barriers between services and older adults. Furthermore, the strategy of the “15-min older people care service circle” should be further implemented by widening the service coverage and shortening the service distance. Additionally, a differentiated pricing strategy can be carried out with multi-grade service, and the government-paid service or welfare voucher should cover the vulnerable older adult groups. Finally, more emphasis should be placed on the high-quality development of CCSs to reinforce the competitiveness of these services, especially for healthcare services. For the government, incentive policies should be implemented to attract professional service institutions as service providers. Additionally, they can conduct regular assessments and set specialized fiscal appropriations to support and encourage the high-quality service providers.

## Limitations

The limitations of this study should be acknowledged. First, although contextual-level factors were considered, they were measured at the individual level due to data limitations. Further research could use multilevel multinomial logistic regression models to address the potential bias arising from between-group variation. Second, due to the limitations of time, funds, and data collection, this study was only based on the cross-sectional survey data and lacked long-term observation and comparison of the transformation of willingness and behavior. In the next step, the tracking survey data will be used to make further analyses from a dynamic perspective. Third, given the limited availability of data, the proportion of the empty-nest older adults was 68.61%, which may lead to over-representation issues. Variables like traditional old-age care beliefs may have insufficient reliability with the single-item measurement. Further studies will improve the comprehensiveness of the survey design and adjust the sampling method to reduce selective bias.

## Conclusion

This study reveals the gaps between willingness and behavior of older adults to use CCSs in urban China and further identifies key factors that convert the willingness into actual behavior. The findings highlight the significant effect of a series of predisposing factors, enabling factors, and needs factors on arousing the willingness of older adults to use CCSs. Among them, age, sex, marital status, educational level, traditional old-age care belief, neighbor’s attitude, policy advocacy, service affordability, province, health status, and empty nest were identified as the key factors to convert the willingness into behavior. On the basis of these findings, targeted policy implications were offered to bridge the willingness-behavior gaps regarding using CCSs among older adults.

## Data Availability

The datasets presented in this article are not readily available because the data are not publicly available due to privacy or ethical restrictions. Requests to access the datasets should be directed to Lijian Wang, wanglijian2@mail.xjtu.edu.cn.
